# Heterogeneous Presentation of Hereditary Neuropathy With Liability to Pressure Palsies: Clinical and Electrodiagnostic Findings in Three Patients

**DOI:** 10.7759/cureus.32296

**Published:** 2022-12-07

**Authors:** Lisa B Shields, Vasudeva G Iyer, Yi Ping Zhang, Christopher B Shields

**Affiliations:** 1 Neurological Surgery, Norton Neuroscience Institute, Norton Healthcare, Louisville, USA; 2 Neurology, Neurodiagnostic Center of Louisville, Louisville, USA

**Keywords:** ultrasound, electromyography, nerve conduction studies, hereditary neuropathy with liability to pressure palsies, neurology

## Abstract

Hereditary neuropathy with liability to pressure palsies (HNPP) is characterized by the acute onset of focal sensory and/or motor deficits when the peripheral nerves are stressed by a mechanical force. Caused by a deletion in the *PMP22 *gene, this condition is often underdiagnosed or misdiagnosed due to the heterogeneity of clinical and electrophysiological presentation. This case series describes the clinical and electrodiagnostic (EDX) features of three patients presenting with clinically heterogenous phenotypes of HNPP. A retrospective analysis of patients referred to our facility for EDX studies identified three patients with genetically confirmed HNPP. The clinical presentations were unique in each patient, and the referring physicians did not consider HNPP in their differential diagnosis. The patients underwent comprehensive clinical and EDX evaluations, which led to the diagnosis of HNPP with subsequent confirmation by genetic studies. The reasons for referral for EDX studies were foot drop in one patient, carpal tunnel syndrome (CTS) in another, and wrist drop in the third patient. Patient #1 was initially diagnosed with chronic inflammatory demyelinating polyradiculoneuropathy (CIDP) at another facility and developed a foot drop while recovering from a cervical discectomy and fusion. A positive family history of neuropathy led to the suspicion of hereditary neuropathy, and further studies confirmed a *PMP22* gene deletion. Patient #2 gave a typical history suggestive of CTS. EDX studies showed focal slowing of conduction at potential areas of entrapment/compression. A history of foot drop in the patient and many other family members led to the confirmation of HNPP by genetic testing. US studies aided in confirming CTS by documenting a significant drop in the diameter of the median nerve within the carpal tunnel. The third patient developed radial nerve palsy after using axillary crutches. The possibility of HNPP was considered due to the pattern of nerve conduction slowing and a family history of foot drop. Physicians should be cognizant of the varied presentation of HNPP and the potential for misdiagnosis and underdiagnosis of this condition. In each case, elicitation of a detailed family history led to the suspicion of HNPP. A high index of suspicion is necessary for patients with multiple episodes of compressive neuropathies, mono/multi mononeuropathies, unexplained polyneuropathy, and a family history of neuropathy. The EDX pattern in patients presenting with acute focal neuropathies, consisting of a global increase in distal motor latencies, moderately decreased motor nerve conduction velocities, and focal slowing of conduction in potential sites of entrapment, should trigger genetic testing for HNPP.

## Introduction

Peripheral myelin protein-22 (PMP22) is mainly expressed in compact myelin of the peripheral nervous system and is an integral membrane glycoprotein of internodal myelin [1,2]. Conditions associated with PMP22 gene mutations disrupt myelin organization and axonal integrity. With a prevalence of 40 in 100,000 people, autosomal dominant Charcot-Marie-Tooth disease is caused by a heterozygous duplication of chromosome 17p11.2-12 and is the leading inherited peripheral nerve disorder [2,3]. With a much lower prevalence (7.3 to 16 per 100,000 individuals), hereditary neuropathy with liability to pressure palsies (HNPP) is due to a heterozygous deletion of chromosome 17p11.2 [4]. Usually affecting individuals in the second or third decade, HNPP is marked by episodic, painless, recurrent focal motor and sensory peripheral neuropathy with sensory (numbness) and motor (muscular weakness and atrophy) findings resulting from involvement at potential sites of compression or entrapment such as the median nerve at the wrist, the ulnar nerve at the elbow, and fibular nerve at the fibular neck [2,4].

When patients present with foot drop, wrist drop, or other mononeuropathies, it is important to look for a history of similar episodes and similar occurrences in other family members. Electrodiagnostic (EDX) studies play a valuable role in diagnosing HNPP as they often demonstrate increased distal motor latencies, prolonged F-wave latencies, and focal slowing of conduction velocities at sites of compression and are normal in other nerve segments [2,4,5]. Sensory nerve conduction velocities are often decreased, and sensory nerve action potential amplitudes are usually reduced. A total of 60%-80% of patients with characteristic EDX findings have HNPP confirmation with genetic analysis [2]. Other modalities that may be beneficial in diagnosing HNPP include an ultrasound (US) (showing increased nerve cross-sectional area [CSA] at sites of compression such as wrist [median], elbow [ulnar], and knee [peroneal] and not at non-entrapment sites) and nerve biopsy (revealing tomacular or “sausage”-shaped folded structures of the myelin sheaths with segmental de- and remyelination and varying large-fiber loss) [1,2,4-6]. 

We present three cases of HNPP with heterogeneous clinical and electrophysiological findings. The physical examination, as well as the EDX and US findings, are discussed. The differential diagnosis and management of HNPP are also highlighted. 

## Case presentation

Case one

History and Physical Examination

A 47-year-old male (BMI: 29.53 kg/m^2^) presented with a three-week history of right foot drop that began while he was recovering from an anterior cervical discectomy and fusion from C4 through C7. The patient had a nine-year history of polyneuropathy. He had a previous episode of foot drop on the right side two years earlier, which improved after 2-3 months. The patient was initially diagnosed with chronic inflammatory demyelinating polyradiculoneuropathy (CIDP) and received intravenous immunoglobulin (IVIG) treatment without benefit. His mother and maternal grandfather had been diagnosed with polyneuropathy, although the cause was not determined.

There was wasting and weakness of the distal muscles in both upper extremities with decreased sensation up to the proximal forearms (Table 1).

**Table 1:** Clinical features of patients with hereditary neuropathy with liability to pressure palsies in our case series R: Right, L: Left, LE: Lower extremities, APB: Abductor pollicis brevis, ADM: Abductor digiti minimi, FDI: First dorsal interosseous, Dec: Decreased

Patient #	Side	Motor Complaints	Sensory Complaints	Muscle Weakness	Sensory Loss	Reflexes
1	R, L	R foot drop	Numbness of hands and feet	Wasting and weakness R, L distal muscles UE; dorsiflexors/ evertors R ankle; clawing of toes R, L	Decreased pain and light touch in R, L hands and distal forearm; feet and distal leg loss of pain and touch; loss of position and vibration sense	R, L knee/ankle reflexes absent
2	R, L	Weakness of right hand. Drops objects held in R hand	Painful paresthesia of hands R>L with nocturnal exacerbations	Wasting and severe weakness of R. APB Mild weakness of FDI, ADM in R, L	Loss of pain and touch sensation over all digits in R, L hands	Normal upper extremity reflexes; dec ankle reflexes R, L
3	R	R wrist drop	None	Weakness of all muscles innervated by R radial nerve	R dorsal forearm	R triceps absent

The dorsiflexors and evertors of the right ankle were markedly weak and were normal on the left. Patellar and Achilles deep tendon reflexes were absent bilaterally. Pain sensation was absent, extending from the feet to the proximal third of the legs. Vibration and position sense were also absent on the right foot. Clawing of the toes was observed bilaterally. 

Electromyography/Nerve Conduction Velocity (EMG/NCV) of the Lower Extremities

Compound muscle action potentials (CMAP) could not be recorded over the extensor digitorum brevis (EDB) on stimulation of the peroneal nerve bilaterally. The tibial nerves showed decreased motor conduction velocity. No sensory nerve action potentials (SNAP) could be recorded on stimulation of the plantar, superficial peroneal, or sural nerves. Needle EMG revealed fibrillations and positive waves in the right tibialis anterior and peroneus longus with no motor units; the short head of the biceps was normal. The left tibialis anterior and peroneus longus did not show denervation changes. These findings suggested an acute right peroneal nerve neuropathy at the level of the popliteal fossa as well as severe sensorimotor polyneuropathy involving the lower extremities.

EMG/NCV of the Upper Extremities

In view of the findings suggestive of motor sensory polyneuropathy in the lower extremities, the upper extremities were also studied. Both median nerves showed marked prolongation of transcarpal motor latency with significant slowing of motor conduction velocity in the forearms (Table 2).

**Table 2:** Electrodiagnostic findings of patients with hereditary neuropathy with liability to pressure palsies in our case series R: Right, L: Left, DL: Distal latency in milliseconds, MNCV: Motor conduction velocity in meters per second, NR: No response

Patient #	Median Nerve DL (R/L)	Median Nerve MNCV (R/L)	Ulnar Nerve DL (R/L)	Ulnar Nerve MNCV (R/L)
1	9.2/9.0	37.0/40.9	6.9/6.7	46.3, 41.6/39.2, 28.5
2	NR/11.4	NR/45.2	3.5/3.4	60.6, 33.3/58.8, 32.0
3	4.8/4.8	50.0/55.5	4.2/4.4	45.1, 43.7/49.0/36.6

The amplitude of CMAP over the abductor pollicis brevis (APB) was decreased. Both ulnar nerves demonstrated significant slowing of motor conduction velocity across the wrist, forearm, and elbow. No SNAP of the median, ulnar, or superficial radial nerves could be recorded bilaterally. Needle EMG showed decreased motor unit recruitment and increased polyphasic motor units diffusely in the upper extremity muscles. 

Diagnosis and Family History of HNPP

This patient was diagnosed and received treatment for CIDP, although without much benefit. The occurrence of two episodes of foot drop, EDX findings suggestive of motor sensory polyneuropathy, peroneal nerve palsy, and family history of polyneuropathy raised the possibility of HNPP. The diagnosis was subsequently confirmed by the documentation of a *PMP22* gene deletion.

Follow-Up

Five years after the initial EDX studies, the patient continued to have significant weakness of the distal muscles of both upper and lower extremities with bilateral wrist drop and foot drop. Deep tendon reflexes were hypoactive in the upper extremities. Pinprick, light touch, and position sense were impaired. Three years later, the upper and lower extremities were increasingly weak, with worsening wrist drop and balance. 

Case two

History, Family History, and Physical Examination

A 51-year-old woman (BMI: 36.09 kg/m^2^) presented with a several-year history of numbness and aching of the hands with nocturnal exacerbations of symptoms. She was a cake decorator and spent a considerable time painting. She was diagnosed with CTS and wore a wrist splint with minimal benefit. Her history was significant for the occurrence of foot drop after childbirth which improved after several months. Several family members, including her father and grandfather, experienced foot drop, and some had foot deformities. Her daughter was later confirmed to have HNPP by genetic testing.

There was significant wasting of the APB on the right side (Table 1). The abductor digiti minimi (ADM), first dorsal interosseous (FDI), and flexor digitorum profundus (FDP) were weak bilaterally. Decreased pinprick sensation was noted over all digits bilaterally. Deep tendon reflexes were hypoactive on both sides. 

EMG/NCV of the Arms

Stimulation of the right median nerve did not evoke CMAP over the APB and second lumbrical muscles (Table 2). The left median nerve revealed marked prolongation of transcarpal motor latency with decreased motor conduction velocity in the forearm segment. No sensory potentials could be recorded on stimulation of the median nerve bilaterally. Stimulation of the ulnar nerves showed slowing of motor conduction across the elbow bilaterally. The CMAPs did not show significant dispersion with proximal stimulation. Needle EMG demonstrated denervation changes in the right APB with no motor units. The left APB was normal. The FDI showed decreased motor unit recruitment and increased polyphasic motor units bilaterally. 

**Figure 1:**
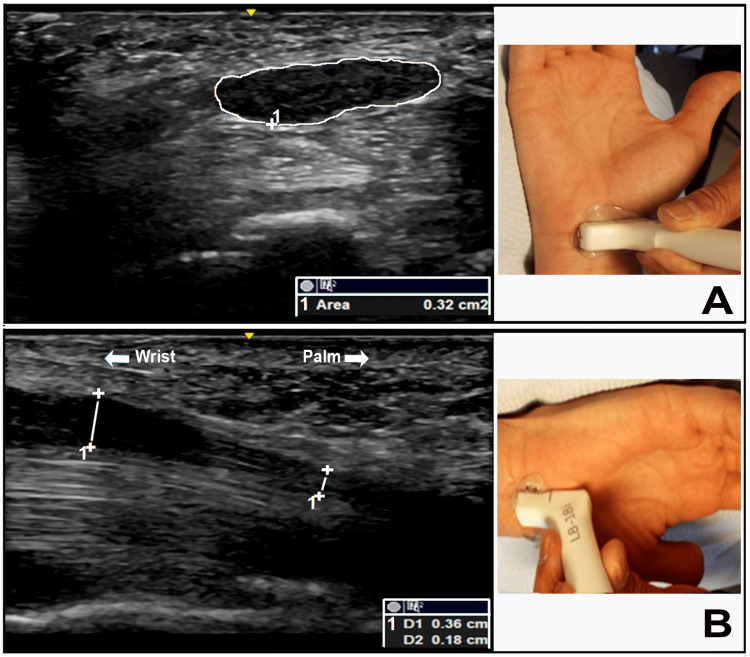
Ultrasound of the median nerve at the carpal tunnel inlet and wrist (A) Ultrasound short axis view of the right median nerve at the carpal tunnel inlet showing a significant increase in cross-sectional area (0.32 cm^2^) compared to the upper limit of normal (0.12 cm^2^). (B) Ultrasound long axis view of the right median nerve at the wrist showing a drop in diameter (left vertical line: 0.36 cm, right vertical line: 0.18 cm) within the carpal tunnel.

The EDX studies suggested a severe median nerve neuropathy on the right, but accurate localization was lacking. The findings pertaining to the left median nerve can be explained based on the presence of a diffuse demyelinating neuropathy or by focal slowing at the carpal tunnel along with retrograde slowing from longstanding entrapment at the carpal tunnel. The findings also suggested the additional presence of a bilateral ulnar nerve neuropathy at the elbow with focal demyelination. Since the EDX study findings did not precisely localize the site of the median nerve neuropathy, the patient underwent an US study using a GE LOGIQ machine (GE Healthcare; Chicago, Illinois) with a linear array transducer of 8-18 MHz, according to our lab protocol [7]. The US showed a marked increase in CSA of the median nerve at the carpal tunnel inlet and a significant drop in diameter within the carpal tunnel (Figures 1A, 1B).

While the EDX and the US findings confirmed right median nerve entrapment at the carpal tunnel, there was still the question of an underlying neuropathy, HNPP. The patient subsequently had genetic testing that confirmed a *PMP22* gene deletion. 

Surgical Intervention

One month following the neurodiagnostic evaluation, the patient underwent a right carpal tunnel release (CTR) and a right cubital tunnel release simultaneously. 

Case Three

History, Physical Examination, and Radiological Imaging

An 18-year-old male (BMI: 25.1 kg/m^2^) presented with a one-week history of sudden onset of right wrist drop. He was using crutches after undergoing surgery for a middle cruciate ligament tear at the knee. On exam, significant weakness of all muscles innervated by the right radial nerve, including the triceps, was noted (Table 1). Pinprick sensation was decreased over the dorsal aspect of the right forearm. The right triceps tendon reflex was absent. A right brachial plexus MRI without Gadolinium contrast was normal.

EMG/NCV of the Arms

The right median nerve showed prolongation of the distal motor latency with decreased motor conduction velocity in the forearm segment; the SNAP showed prolonged latency (Table 2). The right ulnar nerve demonstrated a prolonged distal motor latency and decreased motor conduction velocity across the foreman and elbow; the SNAP showed prolonged latency and decreased amplitude. The left median nerve distal motor latency was also prolonged with normal motor conduction in the forearm. The distal motor latency of the left ulnar nerve was prolonged with decreased motor conduction velocity across the elbow. SNAP over the superficial radial nerve showed a low amplitude. 

Needle EMG revealed no motor units in the right brachioradialis, extensor digitorum communis (EDC), extensor carpi radialis longus (ECRL), and extensor indicis (EI); a single motor unit was recorded in the triceps. 

Family History

The patient’s maternal aunt accompanied him to the neurodiagnostic evaluation. She disclosed that she had experienced episodes of wrist and foot drop. EDX studies on the aunt revealed prolonged distal motor latency and low motor conduction velocity of the right median nerve forearm segment; sensory potentials showed prolonged latency and decreased amplitude. There was also a slowing of motor conduction of the right ulnar nerve across the elbow. The clinical EDX findings and family history suggested the possibility of HNPP, which may have been predisposed to radial nerve palsy due to pressure from the crutches. The underlying pathology was most likely a conduction block in the right radial nerve at or proximal to the innervation of the triceps. A genetic test for PMP22 deletion was subsequently performed, which confirmed HNPP. 

Follow-Up

The patient underwent occupational therapy with electrical stimulation to the affected muscles and continued to wear the wrist brace. Evaluation five weeks later demonstrated greatly improved muscle strength in his right arm, although the triceps and wrist extensors were still weak. There was an increase in motor unit recruitment in the muscles innervated by the right radial nerve compared to the previous study, indicating significant improvement. 

## Discussion

HNPP is often underdiagnosed or misdiagnosed due to the heterogeneity of the clinical and electrophysiological presentation [8]. Recurrent, often painless episodes of mononeuropathies are a common feature of HNPP. The focal deficits are triggered by physical activity (stretching and repetitive motions of the affected limbs) or extrinsic compression and are believed to be due to a mechanically-induced reversible conduction block, specifically, a failed propagation of action potentials along myelinated nerve fibers [1]. Using axonal excitability techniques, Farrar and colleagues investigated the pathophysiological mechanisms associated with HNPP by stimulating the median motor and sensory axons at the wrist [9]. These authors suggested that axonal hyperpolarization in both motor and sensory axons may contribute to the changes in nerve architecture and that the hyperpolarized resting membrane potential in HNPP may be a significant predisposing factor for developing conduction block with mechanical stresses [9].

In addition to the common phenotype of episodic painless focal neuropathies, Mouton and colleagues described five other clinical presentations: recurrent short-term positional, progressive mononeuropathy, CMT-like polyneuropathy, chronic sensory polyneuropathy, and CIDP-like, recurrent subacute polyneuropathy [10]. Taioli and colleagues suggested that micro-mutations of PMP22 cause a clinical and pathological continuum of demyelinating neuropathies that may include atypical phenotypes [11]. 

Each of the cases in our case series illustrates phenotypical heterogeneity of HNPP, which may have caused the referring physician to miss the diagnosis. Patient #1 illustrates the situation where the patient was initially diagnosed and treated with IVIG for CIDP without benefit. This problem has been previously reported in the literature. Beydoun and colleagues reported two patients who were initially diagnosed and treated for an acquired demyelinating disorder or alternative inherited demyelinating disorder [8]. Upon repeat EDX studies and genetic testing, these patients were accurately diagnosed with HNPP. Distinguishing between HNPP and CIDP is important as the treatment for these conditions is markedly different. Patients with CIDP are often managed with IV or subcutaneous immunoglobulin, corticosteroids, plasma exchange, and newer immunomodulating drugs in refractory cases [12,13]. Recent reports have made the situation murkier due to the overlapping symptoms and concurrent occurrence. Shah and colleagues described a patient in whom CIDP manifested as neuropathy with liability to pressure palsies and was successfully treated with IVIG [14]. Vrinten and colleagues reported a patient in whom HNPP coexisted with inflammation which responded to immunomodulatory treatment [15]. Liew and Lo described a case of HNPP and painful neuropathy in which the pain was relieved by IVIG [16]. Treatment for HNPP is usually symptomatic and commonly involves physical and occupational therapy to improve fine and gross motor skills, bracing (wrist splint, ankle-foot orthosis), and avoiding activities that pose a risk for pressure palsies such as prolonged sitting with the legs crossed, repetitive movements of the wrist, prolonged leaning on the elbows, and rapid weight loss [4]. The acute neuropathic symptoms associated with HNPP usually resolve within days or weeks. Half of the patients attain a complete recovery, while 10%-15% experience chronic motor deficits following nerve palsies [4].

Patient #2 in our case series presented another unique problem: how to conclusively diagnose CTS in patients with HNPP. EDX studies may not be conclusive in patients with HNPP who show slowing of sensory and motor conduction in median nerves, findings similar to entrapment at the carpal tunnel. US studies may also not provide the answer, as an increase in CSA of nerves at potential entrapment sites is typically seen in HNPP [6,17]. In this patient, we relied upon the finding of a significant drop in the diameter of the median nerves within the carpal tunnel (Figures 1A, 1B). 

US imaging plays an important role in differentiating between HNPP and other demyelinating polyneuropathies, compression neuropathies, and nerve injuries through pattern recognition (US pattern sum score, homogeneity, and entrapment score) [18,19]. High entrapment ratios (wrist-to-forearm ratio of the median nerve and cubital tunnel-to-upper arm ratio of the ulnar nerve) have been observed in patients with HNPP compared to normal nerve segments between entrapment sites [19]. Furthermore, US is a suitable tool when detecting conditions with non-uniform nerve enlargement [6]. The role of CTR in patients with HNPP is unpredictable [20]. Recovery after CTR is associated with remyelination or nodal reconstruction instead of axonal regeneration, and it is unknown whether PMP22 deletion in HNPP interrupts myelin or nodal reconstitution [20]. Earle and colleagues reported two patients with HNPP who underwent a CTR, both of whom experienced clinical and electrophysiological improvement postoperatively [20]. These authors suggest that patients with HNPP retain the capacity for conduction repair. Patient #2 in our case series underwent both a simultaneous right CTR and right cubital tunnel release. Of note, the potential for pressure palsy is high in patients with HNPP; thus, special precautions such as protective padding over nerves vulnerable to extrinsic pressure were necessary during anesthesia/sedation.

Patient #3 presented with radial nerve palsy after the use of crutches. The only reason to suspect HNPP was the history of foot drop in several family members and the presence of more diffuse nerve conduction abnormalities.

## Conclusions

Physicians should be alert to the varied presentation of HNPP and the potential for misdiagnosis and underdiagnosis of this condition. A high index of suspicion is warranted in patients with multiple episodes of compressive neuropathies, mono/multiple mononeuropathies, and unexplained polyneuropathy. Our cases also highlight the importance of taking a detailed family history for neuropathy. The EDX pattern in patients presenting with acute focal neuropathies, consisting of a combination of a global increase in distal motor latencies coupled with moderately decreased motor nerve conduction velocities and focal slowing of conduction in potential sites of entrapment, should trigger genetic testing for HNPP.
